# Granuloma faciale: clinical, morphological and immunohistochemical aspects in a series of 10 patients*

**DOI:** 10.1590/abd1806-4841.20164628

**Published:** 2016

**Authors:** Cristiano Claudino Oliveira, Pedro Eugênio de Carvalho Ianhez, Silvio Alencar Marques, Mariângela Esther Alencar Marques

**Affiliations:** 1 Universidade Estadual Paulista "Júlio de Mesquita Filho" (Unesp) – Botucatu (SP), Brazil; 2 Hospital Ipiranga – São Paulo (SP), Brazil

**Keywords:** Immunohistochemistry, Inflammation, Pathology, Skin, Vasculitis

## Abstract

Granuloma faciale is a chronic, benign, cutaneous vasculitis with well-established clinical and morphological patterns, but with an unknown etiology. This study describes clinical and pathologic aspects of patients diagnosed with granuloma faciale. The authors analyzed demographic, clinical, morphological and immunohistochemical data from patients with a final diagnosis of granuloma faciale, confirmed between 1998 and 2012. There was a proportional and mixed inflammatory infiltrate, Grenz zones were present in almost all the samples. Immunophenotyping confirmed a higher intensity of T lymphocytes than B lymphocytes in thirteen samples, with a predominance of T CD8 lymphocytes in 64% of cases, in contrast to the literature, which indicates that the major component is T CD4 lymphocytes. All cases were positive for IgG4 but the majority (12/14) had less than 25% of stained cells. The pathogenesis of granuloma faciale remains poorly understood, making studies of morphological and immunohistochemical characterization important to better understand it.

## INTRODUCTION

Granuloma faciale is a chronic, benign, cutaneous vasculitis with well-established clinical and morphological patterns, but with an unknown etiology.^1,2^ The scientific literature on this disease is composed predominantly of case reports. The most recent case series were published by Casinaro *et al.* (2013, 25 patients), Ziemer *et al* (2011, 41 patients), Ortonne *et al.* (2005, 66 patients) and Marcoval *et al.* (2004, 11 patients)^3-6^, while the Brazilian literature lacks granuloma faciale case series.

Studying clinical and histopathological manifestations may be a way to clarify the variables that influence the disease's onset and course. This study discusses cases of Brazilian patients diagnosed with granuloma faciale at a health referral service, characterizing the clinical, epidemiological, histopathological and immunohistochemical features of the lesions.

## METHODS

This is a descriptive, cross-sectional and retrospective study, developed at the Department of Pathology, *Universidade Estadual Paulista "Júlio de Mesquita Filho"*, Botucatu School of Medicine (Unesp). It was approved by the local Ethics Committee.

The histopathology reports file was reviewed for the period encompassing January 1998 (the beginning of digital files) to April 2012, and ten patients with a histological diagnosis of granuloma faciale were identified.

Patients diagnosed with granuloma faciale during this period had their lesion biopsies reviewed by two pathologists. The samples were characterized by lesion location and the specific morphological patterns described in the literature. The inflammatory cell population, analyzed by Hemotoxylin & Eosin (H&E) staining, was quantitatively measured using a grading scale as specified cellularity: from 0 to 25%, 26% to 50%, 51% to 75% and 76% to 100%.

The characterization of the immunophenotypic profiles in inflammatory processes was performed by immunohistochemistry reactions using the following antibodies: CD3 (policlonal, dilution 1:150, source Cell Marque), CD20 (clone L26, dilution 1:250, source Cell Marque), CD4 (clone SP35, dilution 1:100 source Cell Marque), CD8 (clone C8/144B, dilution 1:200, source Cell Marque), immunoglobulin G (IgG) (polyclonal, dilution 1:250, source Cell Marque) and immunoglobulin G4 (IgG4) (clone MRQ-44, dilution 1:250, source Cell Marque). The percentage of cells stained by each antibody was analyzed through the grading scale described above. The cutoff criteria for identifying IgG4-delated disease were: presence of 50 or more stained plasma cells by IgG4 per high-power field (HPF) and an IgG4/IgG ratio higher than 40%.

Demographic data and medical history were collected from medical records, tabulated and submitted for descriptive analysis.

## RESULTS

Between 1998 and 2012, the authors identified 10 patients with granuloma faciale, four of which had had two manifestations in two different years. Most patients (60%) were female and age ranged between 36 and 59 years. Regarding comorbidities, four patients were smokers, and some had hypertension (n = 1), diabetes mellitus (n = 1), dyslipidemia (n = 1), systemic lupus erythematosus (n = 1) and cutaneous malignant epithelial tumors (n = 2). No relationship was found between the lesions and local trauma, though one patient's lesion was near a surgical scar (Table 1).

**Table 1:** Patients diagnosed with granuloma faciale by biopsy between 1998 and 2012

Patient	Number of biopsies	Gender	Age	Comorbidity	Smoker
1	Two	M	54	DM	Yes
2	Two	F	36	Absent	No
3	Two	M	54	NMEC	No
4	One	F	*	Absent	Yes
5	One	M	59	NMEC	Yes
6	Two	F	55	HAS	Yes
7	One	F	41	LES	No
8	One	M	48	Absent	No
9	One	F	*	Absent	*
10	One	F	53	Absent	*

* Information not available; DM=diabetes mellitus; HAS=hypertension; LES=systemic lupus erytematosus; NMEC=non-melanoma cutaneous malignant epithelial tumors.

**Figure 1:**
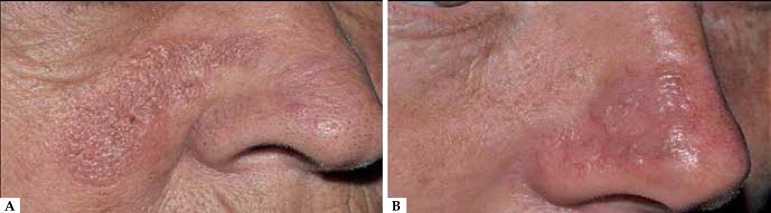
Granuloma Faciale. Clinical photos (A – patient 6; B – patient 2) exemplifying facial involvement by erythematous plaques. In this study, all patients had lesions on the face

All patients had facial lesions, mostly in the nasal, malar and zygomatic regions. The clinical aspect of the lesions varied from macules to papules and plaque, and most patients presented with more than one lesion (Figure 1). Telangiectasis (n = 1) and pruritus ( n = 1) were reported in clinical interviews.

**Figure 2:**
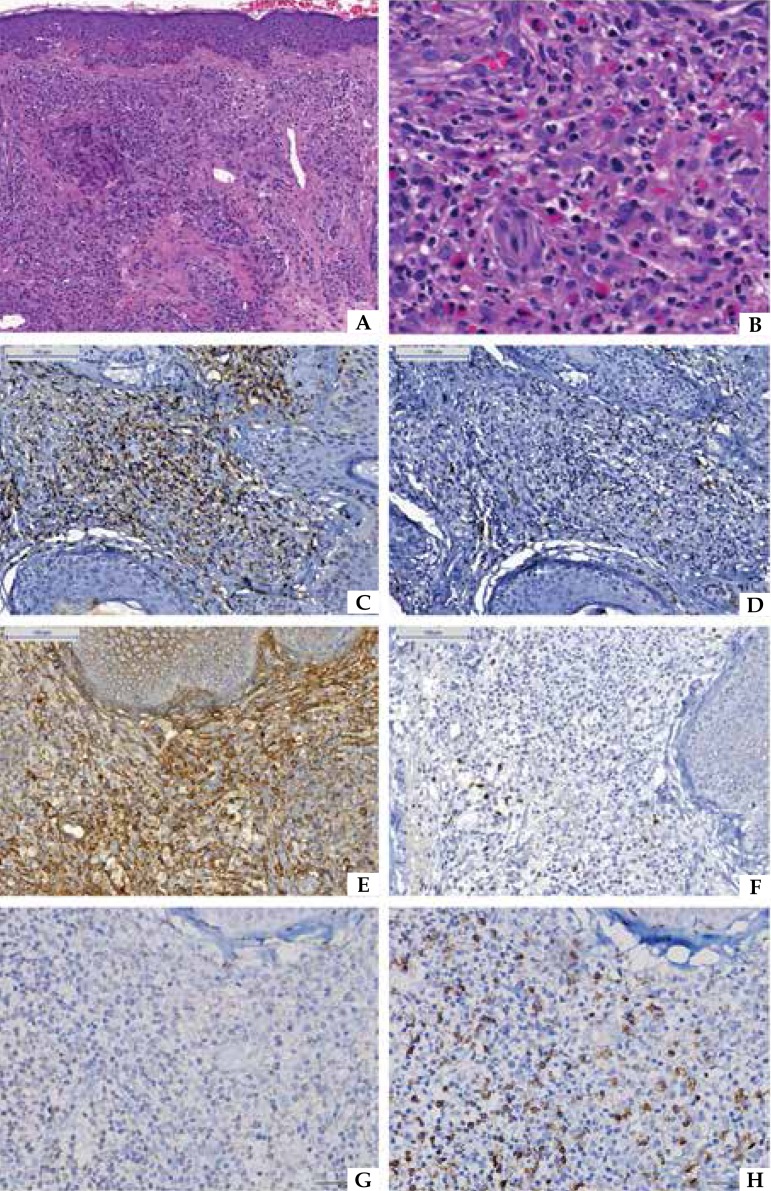
Granuloma Faciale. Pictures A (H&E, 100x) and B (H&E, 200x), showing the Grenz zone and perivascular inflammatory infiltrate composed of neutrophils, eosinophils, lymphocytes and plasma cells. Immunohistochemical images showing a predominance of T lymphocytes (C, CD3, 200x) compared with B lymphocytes (D, CD20, 200x). Pictures E (IgG, 200x) and F (IgG4, 200x) reveal no pattern of IgG4-related disease. There was a higher proportion of T CD8 lymphocytes (H, 200x) than T CD 4 lymphocytes (G, 200x)

Histopathological analysis revealed the presence of a Grenz zone in most samples, both in the epidermis and perifolicular region (12 /14). The irregularity of the perifollicular Grenz zone was also observed in four cases, and in one of them this change was also seen in the epidermis. In all cases, a perivascular mixed inflammatory infiltrate of lymphocytes, plasma cells, eosinophils and neutrophils was observed. Most samples (8/14) had a balanced ratio between cellular inflammatory elements. In other cases (6/14), a more pronounced neutrophil population was noticed (Figure 2). In 13 samples, there was a perivascular inflammatory infiltrate and permeation of the vascular wall, sometimes with fibrinoid degeneration, comprising a vasculitis pattern, typical in granuloma faciale. In one sample, the inflammatory process was diffuse throughout the dermis. A biopsy showed areas of fibrosis adjacent to the perivascular inflammation.

Evaluation by immunohistochemical reaction confirmed a predominance of T lymphocytes compared with B lymphocytes, in 13 samples. The proportion of cells staining by CD3 was higher than 75% in cellularity compared with the CD20 ratio. The population of T lymphocytes was also evaluated by immunostaining for CD4+ and CD8+ cells, indicating a predominance of CD8+ T cells, assessed in 64% of the biopsies. The evaluation revealed positive IgG immunostaining in all cases studied at a variable rate of 25-75% cellularity. IgG4 was expressed by fewer than 50 plasma cells per HPF in all samples (Figure 2). Thus, there were no criteria for diagnosing IgG4-related disease in any of the samples.

Seven patients underwent clinical treatment with topical corticoids but only in one case long-teerm resolution was observed. (Table 2). One patient treated with photodynamic therapy (PDT) relapsed after three years and one patient experienced spontaneous resolution.

**Table 2:** Patients diagnosed with granuloma faciale who had undergone clinical treatment

Patient	Drug	Electrocoagulation	Clinical follow-up
1	Topical corticoid + Dapsone	Yes	Relapse after four years
2	Topical corticoid	Yes	Relapse after three years
6	Topical corticoid + Dapsone	Yes	Relapse after two years
7	Topical corticoid	Yes	Clinical success
10	Topical corticoid	No	*

* Information not available.

## DISCUSSION

Although the literature reports that granuloma faciale is more common in males, appearing between the second and seventh decades of life,^1,6,7,8^ our series of 10 patients revealed a predominance of women, while the age range included the interval between the fourth and sixth decades of life. Lesions are described primarily in the regions exposed to light, as plaques or papules, single or multiple, usually with an erythematous appearance.^1,2,7,8,9,10^ The face is predominantly affected, as observed in this study. ^11^ Extra-facial lesions are reported in the literature, some of them concurrent to the facial lesions.^5,7,11^ In a study by Thiyanaratnam *et al.* (2009), the authors reported that 90% of the lesions began on the face.^8^ The disease has a slow and progressive course and may provoke symptoms of itching and telangiectasias, evolving with periods of exacerbation and remission. The differential diagnosis includes rosacea, lupus erythematosus, sarcoidosis and infectious granulomatous diseases.^1,2,5,7^

Erythema elevatum diutinum (EED) is an important differential diagnosis for granuloma faciale, especially in its extra-facial presentation. Both lesions are leukocytoclastic vasculitis variants. The main differences between the two entities are clinical, so the atypical locations pose greater diagnostic difficulties. EED manifests with multiple lesions on the extensor surface of the joints. Granuloma faciale manifests in isolation, predominantly on the face. As regards morphology, there is an overlap of histopathological findings. Some authors highlight that EED has a major fibrosis component and that granuloma faciale has more inflammatory infiltrate with the Grenz zone. However, it is known that these changes present in a very similar pattern in two entities, not being sufficient for differentiation.^4^ All the patients in this study had facial lesions. Most samples entailed a typical, perivascular, inflammatory pattern. Fibrosis was detected in only one biopsy.

The etiology of granuloma faciale is still poorly understood.^1,8^ Thiyanaratnam *et al.* (2009) reported sun exposure and previhad a history of local trauma due to surgery to remove skin cancer.

Histopathological examination is an essential feature for diagnosing this entity and it is characterized by a dense, perivascular, mixed, inflammatory pattern, consisting of neutrophils, eosinophils, plasma cells and lymphocytes.^2,7,10,11,12^ CD4 + lymphocytes are referenced as important cells in the pathogenesis of granuloma faciale. Imumnophenotypic analysis reveals a predominance of CD4 + lymphocytes, responsible for producing interferon-gamma, a mediator which acts to express molecules such as ICAM-1 on the surface of keratinocytes, promoting the chemotaxis of lymphocytes.^7,8^ Interestingly, in granuloma faciale lesions, basal keratinocytes do not express ICAM-1, restricting the migration of inflammatory cells into the epidermis, forming the characteristic Grenz zone.^13-15^

The 10 cases discussed in this study exhibited morphological patterns equivalent to the descriptions in the literature, with mixed, diffuse and mainly perivascular inflammatory infiltrate composed of neutrophils, eosinophils, lymphocytes and plasma cells.^2,10,11^ Most patients in this study had balanced populations of these cell types. The Grenz zone is reported in 74% to 100% of cases of facial granuloma.^15^ Immunohistochemical analysis of samples from this study revealed a superiority of CD8+ T cells compared with CD4+ T lymphocytes, differing from the current literature.^3,4,5,6,7,12^

Cesinaro *et al.* (2013) suggested recently that the etiology of granuloma faciale might be linked to the IgG4-related lesions spectrum. These authors studied 25 patients with granuloma faciale and 6 of them met the criteria for IgG4-related disease in their biopsy. The criteria adopted were: the presence of 50 or more plasma cells stained by IgG4 and a IgG4/IgG higher than 40% evaluated in HPF.^3,16^ Immunostaining for immunoglobulins in this small series does not confirm the suggestions proposed by Cesinaro *et al.* (2013).^3^ The positivity criteria reported by the authors were not found in any of the biopsies studied.

Because of the lack of detailed pathological mechanisms, there were no efficient treatment protocols.^10,13,17^ The literature highlights surgical interventions and clinical interventions with drugs, single or combined, or combined with therapies such as electrocoagulation. The most commonly prescribed medications include corticosteroids, antimalarials and dapsone.^9,14^

Recently, the literature has emphasized topical tacrolimus in treating these lesions^8,9,13^, though no patient in the study was subjected to this treatment. Tacrolimus is a macrolide antibiotic used in transplant patients by inhibiting lymphocyte activation through interleukins, with consequent reduction in interferon gamma levels.^13^ Gupta *et al.* (2012) reported a case of therapeutic success after three months of tacrolimus in patients whose treatment with dapsone and corticosteroids had been unsuccessful.^17^

Granuloma faciale is a chronic skin disease with well-established clinical and morphological characteristics, though there is a lack of a full understanding of their pathological mechanisms that compromise the development of effective, therapeutic interventions. This study analyzed fourteen samples from ten patients, documenting a predominant involvement of CD8 + T lymphocytes, a different finding to that described in the literature, allowing for improvement in pathophysiological understanding of this disease.
